# The Neuropathology of Autoimmune Ataxias

**DOI:** 10.3390/brainsci12020257

**Published:** 2022-02-12

**Authors:** H. Brent Clark

**Affiliations:** Department of Laboratory Medicine and Pathology, University of Minnesota Medical School, MMC 76, 420 Delaware St. SE, Minneapolis, MN 55455, USA; clark002@umn.edu; Tel.: +1-612-625-7636

**Keywords:** cerebellar degeneration, paraneoplastic, gluten, Hashimoto

## Abstract

Autoimmune-mediated ataxia has been associated with paraneoplastic disease, gluten enteropathy, Hashimoto thyroiditis as well as autoimmune disorders without a known associated disease. There have been relatively few reports describing the neuropathology of these conditions. This review is an attempt to consolidate those reports and determine the ways in which autoimmune ataxias can be neuropathologically differentiated from hereditary or other sporadic ataxias. In most instances, particularly in paraneoplastic forms, the presence of inflammatory infiltrates is a strong indicator of autoimmune disease, but it was not a consistent finding in all reported cases. Therefore, clinical and laboratory findings are important for assessing an autoimmune mechanism. Such factors as rapid rate of clinical progression, presence of known autoantibodies or the presence of a malignant neoplasm or other autoimmune disease processes need to be considered, particularly in cases where inflammatory changes are minimal or absent and the pathology is largely confined to the cerebellum and its connections, where the disease can mimic hereditary or other sporadic ataxias.

## 1. Introduction

The role of autoimmunity in the pathogenesis of ataxia is multifaceted. There are classical autoimmune disorders that involve the nervous system, such as multiple sclerosis [1], acute disseminated encephalomyelitis [2], Behcet disease [3], and collagen-vascular disorders [4] which, when localized to cerebellar pathways, will induce ataxic symptoms. In this review, the emphasis will be on conditions in which the cerebellar system appears to be a major, but not always an exclusive, target of the autoimmune process. The focus will be on the neuropathological findings in these entities and how those findings may differentiate autoimmune ataxias from ataxias due to other causes.

The causes of autoimmune ataxias are complex and, despite the characterization of many autoantibodies associated with those conditions, the role of those antibodies as exclusive pathogenetic mediators is still uncertain, despite the tendency to categorize the diseases based on their presence. This review will not address the purported pathogenetic mechanisms of individual antibodies in any depth, but the neuropathological studies reviewed here suggest that the pathological changes may not always be due to one specific autoantibody. As is the case for many autoimmune phenomena, the dueling concepts of cellular or cell-mediated immunity versus purely humoral immunity also are in play in this arena.

There are cases included in this review, particularly from earlier reports, in which the diagnosis of an autoimmune ataxia was based on an association with either an underlying malignancy or autoimmune disorder, but no autoantibodies were identified. Therefore, there is the possibility that some of these patients, particularly those in whom there were no inflammatory changes in the brain, may have had a coincidental occurrence of a hereditary or other form of sporadic ataxia in conjunction with a malignant tumor or other autoimmune disorder [5].

## 2. Paraneoplastic Ataxia

The occurrence of ataxia in patients with carcinoma has been noted for nearly a century. Most patients with tumor-associated ataxia have gynecologic carcinomas or small-cell carcinoma of the lung, but occasional patients with Hodgkin lymphoma and other malignancies also may develop this condition [6]. Antibodies to certain defined antigens have been implicated in most cases of paraneoplastic ataxia, but not all cases are explained by the presence of known antigens. In gynecologic malignancies, the most common autoantibody is against Yo [7,8] whereas in small-cell carcinoma the most common autoantibody is against Hu [9]. Other characterized autoantibodies have been implicated in paraneoplastic cerebellar degeneration (PCD), but it is likely that additional, yet-uncharacterized antigenic targets will be discovered in future studies.

In 1951, Brain et al. [10] described the pathology of four new cases and reviewed eight previously published cases of ataxia associated with malignant neoplasms. One patient was found to have ovarian carcinoma at autopsy and had severe loss of Purkinje neurons, degeneration of the posterior spinocerebellar tracts and mild degeneration in the distal corticospinal tracts. There was mild perivascular and/or meningeal lymphocytic infiltration in the cerebellum, medulla, and spinal cord. Two patients had previously undiagnosed small-cell carcinoma of the lung. One had severe loss of Purkinje cells and notable loss of granular neurons, as well as neuronal loss in the dentate nucleus and inferior olives. There was fiber loss in the posterior spinocerebellar tracts and to a lesser extent the posterior columns. Lymphocytic cuffing and microglial nodules were seen in the cerebellum and medulla. The other patient with small-cell carcinoma had milder loss of Purkinje cells and granular neurons, with sparing of the dentate nuclei except for mild perivascular lymphocytic infiltrates. The spinal cord had more pronounced lymphocytic infiltration and microglial nodules with mild degeneration in the posterior spinocerebellar tracts and posterior columns. A fourth patient with rapidly progressive ataxia and dementia had mild loss of Purkinje neurons with no inflammatory changes and no pathology in the spinal cord. There was no clinical history of carcinoma, and the autopsy was limited to the central nervous system, so it is uncertain whether this case was truly paraneoplastic. In another report in 1965, Brain et al. [11] described two additional autopsies on patients with pulmonary carcinoma and ataxia. Both had cerebellar cortical degeneration, but no inflammatory changes were mentioned. The spinal cord was normal in the one patient in which it was examined.

### 2.1. Anti-Yo

In 1983, Greenlee and Brashear [8] described two patients who presented with ataxia and were later found to have ovarian carcinoma. At autopsy, both patients were found to have severe loss of Purkinje neurons with milder loss of granular neurons. No other neuropathology was noted. Of interest, sera from these two patients were found to label Purkinje neurons by immunofluorescence in histological sections from normal human cerebellum. This antibody later was named anti-Yo, or anti-Purkinje cell antibody (APCA-1) and is defined by immunoblot reactivity to 34 kilodalton (kDa) and 62 kDa bands from purified Purkinje cell extracts [12]. The 62 kDa band is the primary target and is the cerebellar degeneration-related protein 2 (CDR2), a regulator of transcription that is expressed in Purkinje cells, some brainstem neurons and spermatogonia. This protein also is upregulated in ovarian and breast carcinomas [13]. Three additional autopsy studies of anti-Yo paraneoplastic ataxia were reported [14], showing cerebellar cortical atrophy with severe loss of Purkinje cells in all patients and a reduction of granular neurons in two. Scattered T-lymphocytes were present in the cerebellar arachnoid, but not in the cerebellar cortex. T-lymphocytes formed mild perivascular infiltrates in the dentate nucleus, which was otherwise normal. Another report from the same group [15] described two additional autopsies on patients with anti-Yo antibodies and ataxia. One had profound loss of Purkinje neurons with no other neuronal loss or inflammation in the cerebellum. Scattered rare CD4-positive lymphocytes and macrophages were present in the dorsal medulla. The second patient had similar cerebellar cortical degeneration as well as active degeneration of the corticospinal, posterior spinocerebellar and cuneate tracts in the spinal cord; the spinal roots were normal. The medulla had mild perivascular lymphocytic infiltrates without neuronal loss. Minimal perivascular inflammation was present in the cerebellum. Pyramidal tract degeneration extended to the medullary pyramids. The inflammatory infiltrates were predominantly T-cells with the intraparenchymal cells being mostly CD8-positive and the perivascular lymphocytes being positive for CD4 or CD3. Macrophage/microglial cells also were present in the areas of lymphocytic involvement. The involvement of the spinal cord in the second case led the authors to invoke other mechanisms in addition to anti-Yo, since the Yo-antigens are not present outside the cerebellum and brainstem.

In another report [16] of anti-Yo paraneoplastic ataxia, inflammatory changes were also identified in the brain from a patient with ovarian carcinoma. There was diffuse cerebellar cortical involvement with near-total loss of Purkinje cells and moderate loss of granular neurons. Inflammatory infiltrates were present in the Purkinje cell layer, the deep molecular layer, and cerebellar white matter, along with microglial activation and expression of major histocompatibility complex, class II antigens in endothelia and microglia. The interstitial infiltrates were primarily CD8-lymphocytes, with scattered lymphocytes positive for CD4, CD19 and CD11 in the perivascular sites. There were mild CD8-positive lymphocytic infiltrates in the cerebrum and midbrain with widespread microglial activation but no neuronal loss.

Brock et al. [17] reported findings from a patient with endometrial carcinoma and a ten-month history of progressive ataxia with anti-Yo antibodies. There was widespread severe loss of Purkinje cells with moderate loss of granular neurons, but no inflammatory changes were seen in the cerebellar cortex. The dentate nucleus had scattered lymphocytic infiltration, microglial activation and focal neuronal loss and gliosis. Microglial nodules with focal neuronal loss and astrocytosis were present in the hypoglossal and basal pontine nuclei.

Storstein et al. [18] described autopsies on two patients with serous adenocarcinomas of the ovary and progressive ataxia with anti-Yo antibodies. Both patients had near-total loss of Purkinje cells and Bergmann gliosis with variable regional severity. There were mild perivascular and intraparenchymal CD8-positive lymphocytic infiltrates in the cerebellum, primarily in the deep white matter and dentate nuclei. The infiltrates did not involve the surviving Purkinje cells. B-lymphocytes and CD4-positive T-lymphocytes were not seen. Both patients had degenerative changes in the posterior and lateral columns of the spinal cord with activated microglia. One patient had mild CD8-positive infiltrates in the medulla, while the other had no brainstem pathology.

### 2.2. Anti-Hu and Other Antigens Associated with Small-Cell Carcinoma of Lung

A second major neuronal antigen associated with paraneoplastic ataxia is Hu [9] and it accounts for approximately one-third of all PCD [19]. Anti-Hu recognizes a neuronal nuclear antigen (HuD) that is uniquely expressed in brain tissue. It has homology to the Drosophila proteins, (embryonic lethal abnormal visual system) (Elav) and Sex-lethal, and is involved in neuron-specific RNA processing [20].

These patients typically have small-cell carcinoma of the lung, which in many cases is clinically inapparent at the time of onset of neurological symptoms. Patients with this autoantibody are more likely to have a wider spectrum of neurological disease, with forebrain or brainstem encephalitis, sensory neuropathy or even motor neuron disease in addition to ataxia. A hallmark of anti-Hu-related paraneoplastic disease is an inflammatory infiltrate.

In an autopsy study of five patients with small-cell carcinoma of the lung and anti-Hu paraneoplastic ataxia [21], most were found to have moderate-to-severe loss of Purkinje neurons as well as involvement of the cerebellar and olivary nuclei. Lymphocytic infiltrates were prominent in cerebellar pathways, but also frequently seen in the forebrain, brainstem, spinal cord and spinal roots and ganglia, often with concomitant loss of neurons or axons. One of the five patients had little or no pathology in the cerebellum and minimal inflammatory infiltration, but was clinically ataxic. The spinal cord was not examined in that patient. Two additional patients with small-cell carcinoma but without anti-Hu antibodies or other identified antibodies had loss of Purkinje cells without significant inflammatory changes. One of those patients also had extensive degeneration of corticospinal tracts and posterior columns in the spinal cord. An eighth patient with small-cell carcinoma and paraneoplastic ataxia was negative for anti-Hu, but positive for anti-CV2, an antibody that recognizes the collapse in response mediator protein 5 (CRMP5) and had extensive Purkinje cell loss in the vermis with mild lymphocytic perivascular cuffing in the dentate, brainstem and spinal cord without obvious neuronal loss. More intense leptomeningeal lymphocytic infiltrates were present over the brainstem, cerebellum, and spinal cord. Another autopsy study of anti-CV2-related ataxia in two patients with small-cell carcinoma [22] revealed severe loss of Purkinje cells with mild but widespread inflammation in the brain without neuronal loss. A third patient in that study had both anti CV-2 and anti-Hu antibodies and had diffuse encephalomyelitis with inflammatory infiltrates.

Pathologically, nearly all cases of PCD secondary to small-cell carcinoma of the lung have conspicuous inflammatory infiltrates, typically in multiple sites in the central nervous system (CNS) beyond the cerebellum. Ataxia is the sole manifestation of CNS involvement in only 20% of patients with anti-Hu, and fewer than half of the patients with anti-Hu have ataxic symptoms [19]. Thus, other neurological manifestations in the cerebral cortex, limbic system, brainstem, or spinal cord frequently take the place of or overshadow ataxic symptoms in this group of patients.

### 2.3. Anti-Ri

Patients with breast carcinoma and paraneoplastic ataxia, frequently in combination with opsoclonus, often have antibodies to the Ri-antigen(s) [23], which targets the RNA-interacting region of (neuro-oncological ventral antigen-1) (NOVA-1), a neuron-specific RNA-binding protein that is differentially expressed in the diencephalon, brainstem and cerebellum [24]. Hormigo et al. [25] described a patient with Ri-positive ataxia, opsoclonus, and mild sensorimotor neuropathy who died three years after onset. No malignancy was identified at autopsy, but the breasts were not thoroughly examined. The cerebellum had severe loss of Purkinje cells in the vermis and hemispheres. Lymphocytes infiltrated both the perivascular and parenchymal compartments in the cerebral cortex, thalamus, basal ganglia, and cerebellar dentate nucleus, sometimes accompanied by microglial nodules. The tegmentum in both the pons and midbrain was particularly involved, with conspicuous neuronal loss. The inflammatory infiltrate had both B-lymphocytes and CD4-positive T-lymphocytes in the perivascular cuffs, while the parenchymal inflammatory cells were mostly CD8-positive T-lymphocytes. Natural killer-cells also were identified in the pons. Complement staining was present in blood vessels, neuropil, and neurons of the heavily affected areas, including some of the few residual Purkinje cells in the cerebellar cortex.

### 2.4. Other Paraneoplastic Autoantibodies

Antibodies to P/Q-type voltage-gated calcium channels (VGCC) have been implicated in paraneoplastic cerebellar degeneration, often in conjunction with Lambert–Eaton syndrome [26,27,28]. Most of the reported patients have had small-cell carcinoma of the lung. A neuropathologic study of three such patients [29] found conspicuous loss of Purkinje cells and Bergmann gliosis without inflammatory infiltrates in the cerebellar cortex. One patient also had perivascular lymphocytic cuffing in the dentate nucleus and the surrounding cerebellar white matter. Therefore, PCD due to small-cell carcinoma of the lung is not always inflammatory.

Rare patients with Hodgkin lymphoma develop paraneoplastic ataxia, which has been associated with two different autoantibodies, mGluR1 [30] and DNER (Tr) [31]. The metabotropic glutamate receptor type 1 (mGluR1) is heavily expressed in the cerebellar cortex, particularly on Purkinje cell dendrites, in relation to both parallel fiber and climbing fiber synapses. While the presence of paraneoplastic mGluR1-autoantibodies likely has a pathophysiological effect on the function of these receptors, an autopsy study of a patient with mGluR1-positive Hodgkin-related paraneoplastic cerebellar degeneration [32] showed decreased density of Purkinje cells, most pronounced in the flocculus, nodulus and anterior lobes. In addition, calbindin immunostaining revealed the dendritic truncation and somatic atrophy of surviving Purkinje cells. No inflammatory reaction was detected. There was no mention of other brain regions being affected. DNER (delta/notch-like epidermal growth factor-related receptor) has been shown to be the antigen for the Tr-antibodies that are associated with Hodgkin lymphoma and paraneoplastic cerebellar degeneration [33]. An autopsy study of one patient with Tr-autoantibodies and PCD [31] found “total” loss of Purkinje cells with mild Bergmann gliosis. Milder neuronal loss and gliosis were present in the dentate nucleus and inferior olivary nuclei. There were rare CD3 and CD68 immuno-positive inflammatory cells present in the cerebellum. No residual lymphoma was identified in the systemic postmortem examination.

Other types of malignancies have been present in association with PCD, sometimes with characterized autoantibodies, such as mGluR1, in a patient with prostatic adenocarcinoma and cutaneous T-cell lymphoma [34]. Not all patients with this antibody and ataxia have a detectable underlying malignancy. Other antigens reported as targets in PCD are protein kinase C-gamma (PKC-gamma) and carbonic hydrase-related protein VIII (CARP VIII), but no pathological studies have been published [19].

### 2.5. Paraneoplastic Ataxia without Demonstrated Autoantibodies

An autopsy study of a patient with T-cell lymphoma and PCD was reported with no characterized autoantibody identified [35]. They found neuronal loss, gliosis, and perivascular lymphocytic infiltrates in the cerebellum, inferior olivary nuclei and hippocampi. The infiltrates were T-lymphocytes by immunohistochemical markers but were not malignant, and no residual lymphoma was identified systemically. Postmortem examination of a patient with squamous cell carcinoma of the lung and PCD who was seronegative for anti-Hu, anti-Yo, anti-Ri, and anti-VGCC found “complete disappearance of Purkinje cells throughout the entire cerebellum with reactive astrocytosis”. There was no evidence of metastatic tumor or encephalitic inflammatory infiltrates in the brain. The interval between the onset of symptoms and death was only ten weeks. No mention was made of other brain regions [36].

In a series of 219 patients with PCD, 18% were found to have none of the circulating autoantibodies typically associated with that syndrome [37]. The most common primary tumor types in that subgroup included carcinomas of the uterus, ovary, breast, and lung, as well as lymphoma in women. Pulmonary carcinomas, both small-cell and non-small-cell, were the most common malignancies in men. No pathologic data were provided.

## 3. Gluten-Related Ataxia

Although the association of neurological symptoms originally was described only in patients with celiac disease, later it has been shown that gluten-sensitivity in the absence of enteropathy also can be associated with neurological disorders. One of the more common clinical findings is that of ataxia, although often in the presence of other neurological deficits.

The first comprehensive report with neuropathological findings was that of Cooke et al. in 1966 [38]. They provided autopsy data on 9 of 16 patients with celiac disease and either ataxia or neuropathy. Six of those patients had some degree of cerebellar involvement, with varying degrees of clinical ataxia. The most consistent findings were subtotal, but often widespread, loss of Purkinje cells with Bergmann gliosis. “Atrophy” of neurons and gliosis also were noted in the dentate nucleus and inferior olivary nuclei in a majority of those with cerebellar pathology. Mild perivascular lymphocytic/macrophage cuffs were noted in the cerebellum and/or brainstem in four of the cases. A spinal cord pathology with varying presence of changes in lower motor neurons and degeneration in the posterior and lateral columns was seen in many of the patients including those with and without cerebellar pathology.

Finelli et al. [39] described a single adult patient who presented with rapidly progressive ataxia but did not carry a diagnosis of celiac disease at the onset of the neurological symptoms. He subsequently developed gastrointestinal disease and was diagnosed with celiac disease by jejunal biopsy. He died 1–2 years after onset of ataxia. At autopsy, there was widespread severe loss of Purkinje cells and variable granular cell loss with the greatest involvement being in the vermis and superior hemispheric lobules. The inferior olivary and dentate nuclei also had severe neuronal loss and gliosis with myelin pallor in the inferior and superior cerebellar peduncles. The neostriatum had severe neuronal loss and gliosis, and there were vacuolar changes in the corticospinal tracts in the spinal cord and in the inferior cerebellar peduncles. The posterior columns and lower motor neurons were unaffected. Inflammatory changes were not described.

Another patient with adult-onset rapidly progressive ataxia in a setting of celiac disease was reported by Kinney et al. [40]. In a thorough autopsy examination, they found widespread severe loss of Purkinje cells with milder but extensive loss of granular neurons and thinning of the molecular layer. The dentate nuclei had severe gliosis but mild neuronal loss. The inferior olivary nuclei had severe neuronal loss and gliosis. The basal pontine neurons were spared. The spinal cord had myelin pallor in the lateral columns and loss of neurons in Clarke’s nuclei at multiple levels. Inflammatory changes were not noted.

Bhatia et al. [41] reported an autopsy on a 44-year-old man with a three-year history of myoclonic ataxia and a diagnosis of celiac disease who died by suicide. His neurological condition deteriorated despite dietary therapy. Postmortem findings in the brain were largely limited to the cerebellum with Purkinje cell loss and Bergmann gliosis, most pronounced in the lateral hemispheres. Granular neurons also were affected in the areas with more severe loss of Purkinje cells. There was gliosis in the dentate nucleus without obvious neuronal loss, and mild neuronal loss was present in the inferior olivary nuclei. The cerebral cortex and basal ganglia were unaffected. Vasculitis was not identified and there was no mention of other inflammatory changes.

In a clinical series of 28 patients with gluten-related ataxia, Hadjivassiliou et al. [42] described autopsy findings in two individuals. In one patient there was widespread but patchy loss of Purkinje cells with gliosis, vacuolation and a diffuse T-lymphocytic infiltrate within the cerebellar white matter and posterior columns of the spinal cord. They described perivascular cuffing by mostly T-lymphocytes with fewer B-lymphocytes and macrophages. Peripheral nerves also had sparse lymphocytic infiltrates. The second patient had no cerebellar changes but had “substantial” degeneration of the posterior columns of the spinal cord. There were no inflammatory infiltrates in the brain or spinal cord, but sparse infiltrates were present in peripheral nerves.

Keller et al. [43] described an unusual presentation of gluten-related neurological disease in an adult patient with amyotrophic motor-sensory neuropathy and ataxia developing 2–3 years after diagnosis of celiac disease. An antemortem cerebellar biopsy revealed a necrotizing vasculopathy/vasculitis with subacute ischemic changes. There were macrophage infiltrates and sparse lymphocytic perivascular cuffing. At the autopsy performed several weeks later, there was diffuse loss of Purkinje cells and neurons of the dentate nucleus as well as focal vasculopathic/ischemic lesions in the cerebellum and particularly in the basis pontis, midbrain, thalamus, and striatum. There were mild T-lymphocytic perivascular infiltrates without vascular mural involvement. The spinal cord had mild degeneration in the posterior and lateral columns, with no mention made of anterior horn neurons. The peripheral nerves were not examined.

Another unusual presentation was described by Dimberg et al. [44]. A 68-year-old woman with a 30-year history of celiac disease rapidly developed abnormal spontaneous movements of the hands and face with mild dysarthria and mild ataxia of gait. She progressed to what was diagnosed as myorhythmia by electromyography and died within four months of onset. At autopsy she had widespread atrophy and neuronal loss in the frontoparietal cortex, hippocampus, and thalamus. The cerebellum had severe loss of Purkinje neurons and some loss of granular neurons. The cerebellar nuclei were not mentioned. There was mild perivascular infiltration of T-lymphocytes in several areas of the cerebral cortex and hippocampus.

Inflammatory changes also were reported by Hadjivassiliou et al. [45] in a 32-year-old woman with a rapidly progressive sensorimotor neuropathy and ataxia with celiac disease. At autopsy, she had loss of Purkinje cells and mild loss of neurons in the inferior olivary nuclei. Inflammatory infiltrates were present in the brain and spinal roots. There were microvascular changes with endothelial swelling in multiple regions of the white and gray matter of the cerebrum, cerebellum, and brain stem, with mild perivascular infiltration by macrophages and T-lymphocytes.

Mittelbronn et al. [46] described a 68-year-old man with a 25-year history of progressive ataxia and cognitive decline who had only mild celiac disease symptomatically and by jejunal biopsy, but did have both IgA and IgG anti-gliaden-antibodies. At autopsy, he had early-stage tauopathy in the basal forebrain cholinergic nuclei and mesiotemporal cortex. The cerebellar system was remarkable for prominent neuronal loss, gliosis and microgliosis in the inferior olivary nuclei and cerebellar cortex (Purkinje and granular cells). There was no comment on the cerebellar nuclei. Notably, the changes in the olives and cerebellar cortex were accompanied by CD8-positive lymphocytes often positive for granzyme-B and perforin. B-lymphocytes, plasma cells and CD4-postive lymphocytes were rare to absent. Other brain regions were devoid of inflammation, and only rare CD8-positive lymphocytes were found in the leptomeninges. These findings are suggestive of a cell-mediated immune response as a major factor in gluten-associated ataxia.

A patient with therapy-responsive ataxia was described by Nanri et al. [47]. She presented with truncal ataxia at age 77 that progressed over several years. She did not have an overt diagnosis of celiac disease, but did have an uncharacterized history of “Basedow’s disease”. Her serology was complicated, with elevated antibodies to gliaden, thyroid peroxidase, in addition to SS-A/Ro and SS-B/La (markers of Sjogren’s syndrome). Treatment with IV Ig resulted in moderate improvement of her ataxia. She died at age 85, and the systemic autopsy found evidence of Sjogren’s syndrome as well as thyroid atrophy. Her cerebellar cortex had mild-to-moderate loss of Purkinje cells with no changes in the afferent or efferent connections of the cerebellum. Inflammatory changes were absent leading to them to deduce that this condition was humorally mediated.

In a recent comprehensive review of the neuropathology of gluten-related neurological disease, Rouvroye et al. [48] demonstrated that, while ataxia is a frequent component of gluten-related autoimmune neurological complications, it is not frequently present in isolation, and in many cases may be entirely absent. Common non-ataxic associations include sensory or sensorimotor neuropathy, myopathy or various manifestations of cerebral involvement including cognitive changes and even myoclonus. In some patients in which ataxic features have been clinically overshadowed by other neurological deficits, cerebellar pathology still is present. Inflammatory infiltrates, mostly T-lymphocytic, have been reported in some autopsy studies, but are not a constant feature, so the role of cell-mediated immunity remains uncertain, although there is some evidence for both cellular and humoral causation.

To summarize the reported neuropathology of gluten-related ataxia, nearly all patients studied at autopsy had significant loss of Purkinje neurons with Bergmann gliosis. The other commonly affected targets were cerebellar granular neurons, inferior olivary neurons, and cerebellar nuclei. In many patients, the cerebellar nuclei were gliotic but neuronal loss was equivocal or absent, a change that could be secondary to the loss of afferent fibers from degenerated Purkinje neurons without primary pathology in the nuclear neurons. Olivary changes could represent retrograde trans-synaptic degeneration. In many reports, there is little or no mention that afferent connections from the spinal cord were studied or found to be affected, and the basal pontine nuclei were spared when commented upon. Inflammatory changes were variable and not always correlated with the chronicity of the disease process. Therefore, when inflammatory changes are absent, it is difficult to separate these cases from other hereditary or sporadic ataxias by neuropathological findings alone. The clinical course also can be more protracted than is usually seen in paraneoplastic autoimmune ataxia.

## 4. Hashimoto-Related Ataxia

Ataxia associated with myxedema had been described in various reports as early as the late 19th century. Whether those patients had autoimmune thyroiditis is uncertain. It has been well-established that Hashimoto thyroiditis is associated with numerous neurological complications, including cognitive disorders, seizures, movement disorders and psychosis, in addition to ataxia. While Hashimoto is usually considered the etiology, other autoimmune thyroid conditions such as Graves’ disease can produce a similar syndrome, leading to the suggestion that this condition be termed “steroid-responsive encephalopathy associated with auto-immune thyroiditis” [49].

In a review of published cases of thyroid-related ataxia, Ercoli et al. [50] found that there were two populations of patients with neurological findings and thyroid disease, one with simple hypothyroidism and one with Hashimoto thyroiditis. Approximately two-thirds of these patients were female, and the patients with simple hypothyroidism tended to have uncomplicated ataxia, whereas the patients with Hashimoto thyroiditis sometimes had additional neurological problems. The patients with simple hypothyroidism had complete or partial improvement of their ataxia with hormone-replacement therapy, while the patients with Hashimoto thyroiditis had similar results after immunosuppressive therapy.

Ataxia is not the most common manifestation of this syndrome, and even when present it may be in conjunction with neuropsychiatric symptoms and seizures. Patients typically have autoantibodies to certain thyroid antigens, thyroglobulin and/or thyroid peroxidase, and the more recently described amino-terminal of alpha-enolase [51]. It is uncertain whether these antibodies are the actual mediators of the syndrome, as not all patients with these antibodies have neurological disease.

Despite the recognition of this entity for many years, there have been surprisingly few neuropathological studies published. The first [52] was reported over a century ago in an elderly woman with myxedema, long-standing dementia and ataxia. At autopsy, there was widespread arteriosclerosis, but specifically there was loss of Purkinje neurons and shrinkage of the granular and molecular layers of the cerebellum with “swollen degenerate” axons with silver staining (axonal torpedoes?). A second report, by Price and Netsky [53], was in a patient with longstanding myxedema and ataxia, but also a history of alcoholism. They found moderate loss of Purkinje cells with focal loss of granular neurons that was most pronounced in the vermis, a pattern also seen in alcoholic cerebellar degeneration. They also found scattered periodic acid-Schiff-positive bodies that resembled corpora amylacea but were degradable by diastase. Neither study commented on inflammation.

The most comprehensive neuropathological study of Hashimoto-related ataxia was published by Barnard et al. [54], describing a 57-year-old woman with a two-year history of progressive gait ataxia as well as some cognitive slowing in a setting of hypothyroidism. She was placed on both immunosuppressive therapy and hormone replacement with improvement of her neurological symptoms. Unfortunately, she had poorly controlled hypertension and died of an intracerebral hematoma one month after starting therapy. The general autopsy had evidence of autoimmune thyroiditis with follicular atrophy and a lymphocytic infiltrate. The brain had mild gross atrophy of the cerebellum, more pronounced in the anterior-superior midline, and shrinkage of the basis pontis. Microscopically, there was Purkinje cell loss with Bergmann gliosis, axonal torpedoes, and empty basket fibers. Occasional Purkinje perikarya were heterotopic in the molecular layer. The granular neurons also appeared to be decreased in density. The dentate nuclei had increased “lipid pigment”, but no neuronal loss was noted, although there was mild perivascular cuffing, presumably by lymphocytes. There was mild gliosis in the cerebellar white matter. The pons had atrophy of the basal pontine nuclei and transverse fibers with obvious loss of volume. The superior and middle cerebellar peduncles were atrophic, with lesser involvement of pontine pyramidal fibers. The pyramidal tracts in the medulla and spinal cord, however, were described as normal. The olivary nuclei were not described as having neuronal loss, but there was mild pallor of hilar fibers. The spinal cord was unremarkable except for “mild peripheral pallor” of myelin staining.

Neuroimaging studies also have been performed in patients with autoimmune thyroid disease and have shown varying amounts of cerebellar atrophy. A report of six patients studied by magnetic resonance imaging (MRI) [55] found cerebellar atrophy in all six, mostly in midline structures, with two patients having either mild or extensive basal pontine atrophy and loss of the “olivary bulge”.

There also is a paucity of pathological descriptions of patients with suspected Hashimoto “encephalitis” without ataxia. Nolte et al. [56] described vasculitic changes in the brainstem, and Duffey et al. [57] found mild but widespread perivascular lymphocytic infiltrates throughout the brain and subarachnoid space in a patient with Hashimoto encephalopathy and status epilepticus. There was also diffuse gliosis of the cerebral cortex, basal ganglia, thalami, and hippocampi, with milder involvement of the cerebral white matter. George et al. [58] found perivascular lymphocytic cuffing in the endomysium of skeletal muscle biopsy from a patient with spastic paraparesis and elevated anti-thyroid antibodies.

Hashimoto-related neurological disease thus remains poorly characterized as a pathological entity. Several studies have shown at least perivascular inflammation that often extends beyond the cerebellar system. Vasculitis has not been a consistent finding. Rare cases have shown pathological or radiographic evidence of involvement of the basal pons, thus mimicking olivopontocerebellar degenerations of the hereditary or sporadic types. Therefore, clinical evidence of anti-thyroid antibodies and/or the presence of inflammatory changes at autopsy would be necessary to raise the possibility of Hashimoto-related neurodegeneration.

## 5. Other Non-Neoplastic Autoimmune Ataxias

There are patients who develop apparent idiopathic ataxia who have expression of nervous-system-directed autoantibodies that are associated with paraneoplastic cerebellar degeneration but have no evidence of an underlying malignancy. Because of the frequently occult nature of the neoplasms that give rise to paraneoplastic cerebellar degeneration, the distinction between paraneoplastic and nonneoplastic autoimmune ataxias can be difficult. When antibodies typically associated with paraneoplastic degeneration are identified, it is often assumed that there is an underlying neoplasm which, unfortunately, is sometimes not discovered, even at postmortem examination.

In a study by Burk et al. [5] eight patients with sporadic late-onset ataxia were found to have antibodies to voltage-gated calcium channels. There was no evidence of neoplasia, but three of the eight had what appeared to be multiple system atrophy of the cerebellar type, with olivopontocerebellar atrophy on MRI. The other five patients had cerebellar atrophy only. No pathological data were available. These findings raise the question of whether antibodies to VGCC are pathogenic or may represent a secondary immune response to post-degenerative presentation of Purkinje cell antigens arising from other mechanisms either hereditary or sporadic.

Anti-GAD (glutamic acid decarboxylase) antibodies were reported in a 66-year-old woman with progressive ataxia and type I-diabetes mellitus without neoplastic disease [51]. She died after a five-year course, which included new symptoms of stiff-person-syndrome. Postmortem examination identified near-total loss of Purkinje cells with diffuse Bergmann gliosis and numerous empty basket fibers. There was no evidence of inflammatory changes in the cerebellum and no degeneration or inflammation elsewhere in the central nervous system, but the pancreas did have insular lymphocytic inflammation and cell loss. Anti-GAD antibodies have also been associated with paraneoplastic ataxia, but typically not as the sole autoantibody.

Other autoantibody targets associated with immune ataxia, usually in a non-neoplastic setting, include Homer-3, ITPR1 (inositol triphosphate receptor), ARGHAP26 (Rho GTPase activating protein), and the glutamate receptor, GluR-delta2. Except for GluR-delta2, these usually result in progressive ataxia with incomplete response to immunosuppressive therapy and evidence of cerebellar atrophy over time on neuroimaging [19]. Pathological studies have not been reported. Balint et al. [59] reviewed a comprehensive list of autoantibodies that have been associated with autoimmune ataxia, often in conjunction with other involvement of the nervous system. No autopsy studies have been performed on any of these although there are several brain biopsy studies of patients with glial fibrillary acidic protein (GFAP)-autoantibodies and neurological disease [60,61,62]. It is not clear whether these biopsied patients had ataxia, and the cerebellar system was not examined. One characteristic finding was perivascular cuffing by B-lymphocytes and plasma cells with parenchymal infiltrates, mostly composed of T-lymphocytes (CD8-predominant) and microglia/macrophages. Loss of astrocytes and neurons along with demyelination was noted in one study, but not seen in another.

Post-infectious cerebellar ataxia usually is seen in pediatric patients, and has an excellent prognosis with full recovery in most instances [63,64]. In one series, the most frequent known antecedent infection was varicella, but other, unspecified, likely viral infections were present in half of the patients. No known prodrome was seen in 19% and 3% were thought to be post-vaccination [63]. In a series of 11 adult patients [65], the antecedent infection was Epstein–Barr virus in four and varicella–zoster virus in two, with five having no viral antibodies identified. The younger patients all recovered, while two of three patients over 60 years of age had persistent ataxia with “infratentorial atrophy” on MRI. Patients with transient disease typically have no changes on MRI. Because of the relative benignity of this condition, neuropathological studies are not available.

In 2008, Hadjivassiliou et al. [66] demonstrated that a significant number of “sporadic” ataxias might be of autoimmune origin, despite having no previously characterized autoantibodies demonstrable or not being associated with paraneoplastic conditions or inflammatory disorders such as Hashimoto thyroiditis or celiac disease. The term primary autoimmune cerebellar ataxia was proposed, largely as a place-holding category until specific autoantibodies can be identified. There is a tendency for patients in this suspected category to have midline cerebellar involvement by symptoms and neuroimaging, at least initially, but postmortem studies specific to this entity have not yet been published.

Conversely, neuropathologists occasionally perform autopsy studies on patients with more widespread inflammatory neurodegenerative pathology that also involves the cerebellar system. These patients may have had clinical manifestations of ataxia, usually admixed with progressive involvement of other regions of the brain, spinal cord, and even peripheral nervous system. While there is a possibility of PCD due to undetected malignant disease in some of the cases, many of these patients have no evidence of a neoplastic process or identifiable autoantigens.

## 6. Summary

Although there are numerous reports of clinical and laboratory studies of autoimmune ataxias, the neuropathological literature is limited to the relatively few investigations summarized in this review. Many of those pathological investigations were not reported with much detail, often commenting only on the cerebellar cortical findings with limited description of their distribution within the cerebellum. Other parts of the cerebellar pathways, such as spinal afferents, inferior olivary nuclei and the cerebellar nuclei and peduncles are mentioned in some accounts as having variable amounts of pathological involvement. The basal pontine nuclei, a site of involvement in many of the more common hereditary spinocerebellar ataxias and in multiple system atrophy, are rarely mentioned, and appear to be infrequently affected in autoimmune ataxias.

It is not unusual for a patient to come to autopsy with a diagnosis of ataxia that is of uncertain origin. Some of these patients can be identified as having hereditary forms, particularly in families with more than one affected individual. Pathological clues and clinical information can increase the likelihood of establishing a diagnosis of autoimmune ataxia. Rapid clinical progression is common in paraneoplastic ataxia, which differentiates it from hereditary or most other sporadic ataxias, but the clinical course is less helpful in gluten-related or Hashimoto-related disease.

The presence of inflammatory infiltrates on histological examination is the most reliable indicator of potential autoimmune etiology, even without a history of known malignancy or systemic manifestations of autoimmunity. Patients with anti-Hu are most likely to manifest inflammatory changes, and in most such patients with PCD the inflammation extends beyond the cerebellar system and the neurological deficits are more diffuse. Patients with anti-Hu typically have small-cell carcinoma of the lung, which may be clinically occult, secondary to the immune reaction to its presence. A full postmortem examination is helpful in establishing the presence of the tumor, but in some cases a neoplasm is not found even with careful examination of the lungs. Patients with CV2-antibodies also tend to have inflammation in many regions of the nervous system, as do patients with anti-Ri. In other types of PCD, however, inflammatory infiltrates are less reliable indicators, but clearly helpful when present. Some patients with anti-Yo have been reported to have mild inflammatory infiltrates, typically not in the cerebellar cortex, but many have no evidence of inflammation. It has been suggested that the lack of inflammation at autopsy might represent a later stage of disease in which the inflammatory changes have dissipated [14]. Likewise, with other PCD-related autoantibodies, such as VGCC, mGluR1 and DNER, there is mild or absent inflammation.

In patients without inflammatory changes, the diagnosis of autoimmune ataxia hinges on serological studies or findings of malignancy antemortem or in the autopsy. In general, the pathology of these non-inflammatory cases tends to be primarily cerebellar cortical, often more severe in the midline structures, but there are reports of more complex pathology as well. Table 1 provides a general outline of possible distinguishing features seen in the differential diagnosis of ataxia at autopsy.

## Figures and Tables

**Table 1:** Comparison of autoimmune, sporadic and hereditary ataxias.

**Paraneoplastic ataxias:** Anti-Yo: ovarian/uterine carcinoma; cerebellar cortical degeneration with rare involvement of brainstem/spinal cord; inflammation mild-to-absent.
Anti-Hu: small-cell carcinoma; widespread CNS inflammation with ataxia and cerebellar degeneration often a lesser feature of CNS involvement.
Anit-CV2: small-cell carcinoma; cerebellar cortical atrophy; mild inflammation.
Anti-Ri: breast carcinoma; ataxia with opsoclonus; degeneration in cerebellar cortex and brainstem; inflammation is widespread including supratentorial areas.
Anti-VGCC: small-cell carcinoma; Purkinje cell loss; mild-to-absent inflammation in cerebellar white matter and deep nuclei.
Anti-mGluR1: Hodgkin lymphoma; Purkinje cell loss; no inflammation.
Anti-Tr (DNER): Hodgkin lymphoma; Purkinje cell loss; secondary changes in Dentate nucleus and olives; minimal inflammation.
No demonstrated autoantibodies: Cerebellar cortical degeneration; variable presence of inflammatory infiltrates in the cerebellar system and often elsewhere in CNS.
**Gluten-related ataxia:** Degeneration of cerebellar Purkinje cells and variably granular neurons; gliosis in dentate and olives; some cases have supratentorial, spinal or peripheral nerve involvement (with or without concomitant ataxia); inflammation is variable and not well correlated with chronicity.
**Hashimoto-related ataxia (anti-thyroid antibodies):** Degeneration of cerebellar Purkinje cells and milder loss of granular neurons; basal pontine and olivary atrophy with sparing of dentate nucleus; minimal perivascular inflammation; other CNS manifestations are common with ataxia not always present.
**Other non-neoplastic autoimmune ataxias:** Anti-GAD: Associated with diabetes mellitus-type-1; severe cerebellar cortical degeneration without inflammation in one case.
Anti-GFAP: Inflammatory changes (CD8-lymphocytes, B-lymphocytes, plasma cells, microglia/macrophages) and loss of astrocytic markers in brain biopsies; cerebellar pathology has not been characterized.
**Sporadic ataxias:** Cerebellar cortical atrophies: Degeneration in Purkinje cells with secondary changes in olives; sparing of deep nuclei; no inflammation.
Olivopontocerebellar atrophies: Degeneration in Purkinje cells, basal pontine nuclei, inferior olivary nuclei with sparing of deep cerebellar nuclei; no inflammation or inclusion bodies.
Multiple system atrophy: Olivopontocerebellar atrophy; sparing of deep cerebellar nuclei; atrophy in sympathetic system with milder atrophy in substantia nigra and putamen, no inflammation but characterized by alpha-synuclein-positive glial cytoplasmic inclusions.
**Inherited ataxias:** Widely variable degenerative changes ranging from cerebellar cortical degeneration to olivopontocerebellar, spinal cord, upper and lower motor neuron, sensory nerve, cranial nerve, extrapyramidal, and cerebral cortical degeneration. No inflammation; some dominantly inherited forms may have ubiquitinated intranuclear inclusions.
